# A stochastic context free grammar based framework for analysis of protein sequences

**DOI:** 10.1186/1471-2105-10-323

**Published:** 2009-10-08

**Authors:** Witold Dyrka, Jean-Christophe Nebel

**Affiliations:** 1 Institute of Biomedical Engineering and Instrumentation, Wroclaw University of Technology, Poland; 2 Faculty of Computing, Information Systems & Mathematics, Kingston University, London, UK

## Abstract

**Background:**

In the last decade, there have been many applications of formal language theory in bioinformatics such as RNA structure prediction and detection of patterns in DNA. However, in the field of proteomics, the size of the protein alphabet and the complexity of relationship between amino acids have mainly limited the application of formal language theory to the production of grammars whose expressive power is not higher than stochastic regular grammars. However, these grammars, like other state of the art methods, cannot cover any higher-order dependencies such as nested and crossing relationships that are common in proteins. In order to overcome some of these limitations, we propose a Stochastic Context Free Grammar based framework for the analysis of protein sequences where grammars are induced using a genetic algorithm.

**Results:**

This framework was implemented in a system aiming at the production of binding site descriptors. These descriptors not only allow detection of protein regions that are involved in these sites, but also provide insight in their structure. Grammars were induced using quantitative properties of amino acids to deal with the size of the protein alphabet. Moreover, we imposed some structural constraints on grammars to reduce the extent of the rule search space. Finally, grammars based on different properties were combined to convey as much information as possible. Evaluation was performed on sites of various sizes and complexity described either by PROSITE patterns, domain profiles or a set of patterns. Results show the produced binding site descriptors are human-readable and, hence, highlight biologically meaningful features. Moreover, they achieve good accuracy in both annotation and detection. In addition, findings suggest that, unlike current state-of-the-art methods, our system may be particularly suited to deal with patterns shared by non-homologous proteins.

**Conclusion:**

A new Stochastic Context Free Grammar based framework has been introduced allowing the production of binding site descriptors for analysis of protein sequences. Experiments have shown that not only is this new approach valid, but produces human-readable descriptors for binding sites which have been beyond the capability of current machine learning techniques.

## Background

From the very beginning of modern biology, which can be traced to the 1950s when the structure of the DNA was unveiled, linguistic metaphors were readily used to describe the molecular world. Linguistics itself experienced a revolution led by Noam Chomsky in the second half of 20th century [1]. In the 1980s his work regarding mathematical theory of language was adopted in the field of molecular biology by several researchers [2-4]. Many similarities between natural languages and the language of nature have been revealed. For instance, any functional polypeptide can be regarded as syntactically proper. However, similarly to natural languages, where a correct grammatical structure does not imply that a sentence is meaningful, not all amino acid chains fold into proteins with physiological functions.

According to the linguistic level of the analysis, computational tools utilised in biochemistry can be divided into those operating at the lexical, structural, semantic and pragmatic levels [5]. The first level is occupied by algorithms providing statistical analysis [6]. Structural or syntax features are examined by software which parse sequences [7,8]. The embedded ambiguity of structures, as well as flexibility and versatility are among the advantages of this approach. Recently, an increasing amount of linguistic methods with a probabilistic component has been investigated at the structural level, i.e. Hidden Markov Models [9] and stochastic Context-Free Grammars [10]. At the semantic level a representation of meaning is assigned to the structure [11] and at the pragmatic level some context of the sequence (e.g. relationships between proteins) is taken into account.

In the field of protein sequence analysis, the size of the alphabet and the complexity of relationships between amino acids have mainly limited the application of formal language theory to the production of grammars whose expressive power is not higher than stochastic regular grammars. The first rules were designed to define short functional patterns consisting of adjacent and well conserved amino acids. They are expressed by non-probabilistic regular grammars, e.g. PROSITE patterns [12] and PRINTS [13,14]. Although their expressive power is fairly limited, they have proved extremely useful in protein annotation and detection of important protein regions (e.g. active sites) by highlighting short sub-sequences associated to biochemical functions. Approaches based on Hidden Markov Models (HMMs) are regarded as the state of the art methods in the field of protein sequence annotation. Specifically, Profile HMMs, which were introduced by Krogh et al. [15], are widely used and proved their efficiency for representing motifs, calculating multiple alignments, and profile analysis [10]. However, an important drawback of HMM profiles is that they are not human-readable and, therefore, these descriptors cannot provide any biological insight by themselves. In addition, since the expressive power of an HMM is similar to a stochastic regular grammar [16-18], they have limitations regarding the types of patterns they are able to encode. For example, they cannot cover any higher-order dependencies such as nested and crossing relationships that are common in proteins, e.g. anti-parallel β-sheets and parallel β-sheets respectively [19]. Similarly, bonds in binding sites often exceed the capability of regular grammars and HMMs [20].

Attempts to produce systems with an increased expressive power have been limited [7,10,21] and, according to our knowledge, the only practical tool dedicated to protein analysis was built using stochastic tree grammars to predict both anti-parallel and parallel β-sheets [36,63]. These weakly context-sensitive grammars could not only predict which amino acids were involved in sheets, but also the locations of the hydrogen bonds. However, the structure of the grammar had to be provided; their algorithm learned the probability parameters.

**Figure 1:**
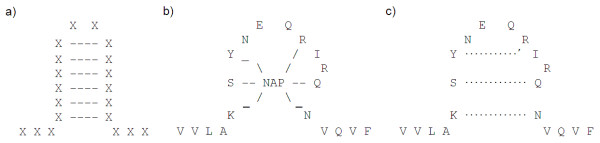
a), b) Typical structures of antiparallel β-sheets (here beta hairpin) and NAP binding region of aldo-keto reductases - here shown on 1MRQ - respectively c) NAP binding site modelled as involving indirect nested dependencies.

Context Free Grammars (CFGs) have the potential to overcome some of the limitations of HMM based schemes since they have the next level of expressiveness in Chomsky's classification and produce human-readable descriptors. Although they do not have the power of context-sensitive grammars and, therefore, cannot deal with crossing relationships, their reduced complexity makes them more practical and allows the possibility of learning grammar structure from examples. Consequently, they could potentially be used to describe a variety of patterns including nested relationships. Anti-parallel β-sheets are natural candidates, see Figure 1a). Moreover, we believe that many ligand binding sites, where main dependencies are essentially branched and nested like, could be detected using CFGs. These relationships are often not direct interactions between amino acids, but indirect through the intermediate of a ligand. For example, the NAP (Nicotinamide-Adenine-Dinucleotide Phosphate) binding region of aldo-keto reductases, see Figure 1b), could be modelled as involving indirect nested dependencies between NAP binding residues, Figure 1c). Moreover, CFGs can be utilised to model dependencies between different parts of a binding site, such as beta strands, helices and loops, by using branching rules. Thus, the development of grammars which have the abilities to model branched and nested relationships should permit to improve modelling of such type of binding sites.

CFG have already been applied successfully in the fields of bioinformatics, particularly for RNA structure prediction [5,10,22-24] and compression [25]. A CFG is particularly adapted to this task because it can express, unlike regular grammar, the nested dependencies due to the Watson-Crick pairing which is key to RNA structure. Due to a larger set of terminals (20 amino acids) and less straightforward relations between residues (there is no equivalence to the Watson-Crick pairing), utilisation of Context-Free Grammars to analyse proteins has not been, so far, comparably successful.

Since the design of an unbiased negative sample is particularly difficult in protein sequence analysis, the fact that CFGs cannot be inferred from positive data only is a serious drawback [26]. An alternative is to develop an approach based on stochastic grammars which, in principle, do not require a negative set for their inference [17,27].

In this paper, a Stochastic Context Free Grammar based framework for the analysis of protein sequences is presented and applied to the interpretation and detection of amino acids involved in binding sites. We start by demonstrating the value of our framework by showing the biological insight which is provided by the produced grammars. Then, we assess its performance in sequence annotation and binding site detection and evaluate them against profile HMMs. In the Methods section, we present the general principles which are behind our framework and the key strategies it relies on. Formal definition of Stochastic Context Free Grammars, implementation aspects and detailed description of datasets are provided in the Appendix.

## Results and discussion

### Description of datasets

We evaluate our framework using a set of binding site patterns which are based on PROSITE entries [56]. They vary in term of size and complexity and include PROSITE patterns and domain profile, as well as a zinc finger 'meta-pattern' that was derived from 7 zinc finger PROSITE patterns (see Table 1). The negative test set is defined as a representative set (up to 30% sequence identity) of all protein sequences available in the Protein Data Bank (PDB) [58].

**Table 1:** Binding site patterns used for evaluation

	**Brief description**
**PS00219**	PROSITE pattern for the anion exchanger family (PDOC00192)

**PS00063**	PROSITE pattern for the aldo-keto reductase family (PDOC00061). Family binds Nicotinamide-Adenine-Dinucleotide Phosphate (NAP).

**PS00307**	Pattern created by extension of PROSITE pattern which is a legume lectin beta-chain signature (PDOC00278). Family binds calcium and manganese located in the C-terminal section of the beta-chain.

**MPI phosphatase**	Pattern created by selecting the fragment which interacts with sulphate ions (SO4) from the PROSITE profile for Rhodanese domain (PS50206) which is present in M-phase inducer (MPI) phosphatase family.

**Zinc finger**	Zinc finger meta-pattern created using 7 different PROSITE zinc finger patterns: PS00028, PS00518, PS00752, PS01030, PS01102, PS13000 and PS01358.

A complete description of these datasets is provided in Appendix E.

### Analysis of grammar structures

The rational for utilising Stochastic Context Free Grammars to produce ligand binding site descriptors is that not only they have the power to express branched and nested like dependencies, but also their rules can be analysed to acquire biological knowledge about binding sites of interest. In this section, we illustrate how analysis of sequence based SCFGs allows gaining an insight into the spatial configuration of binding sites. We propose two ways of analysing probabilistic grammars to extract biologically meaningful features focusing on either parse trees or grammar rules.

**Figure 2:**
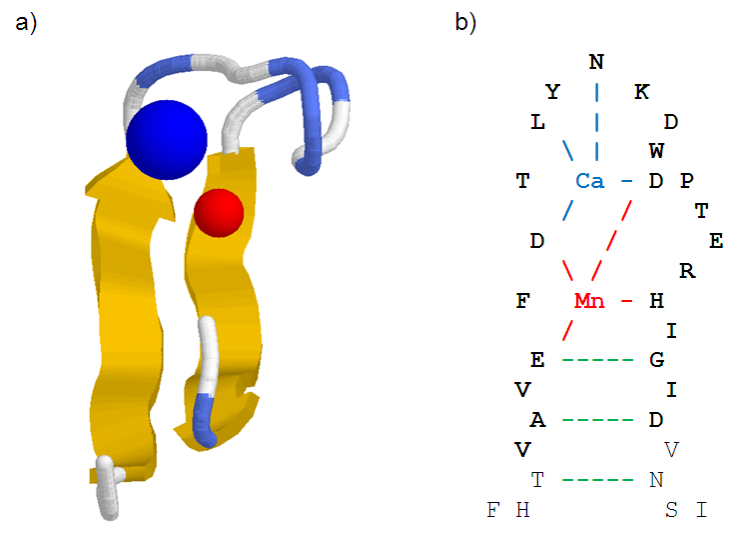
**a) 3D structure of the extended PS00307 pattern in 1FAT b) Hydrogen-bonds involved in ligand binding and beta sheet (residues in bold belong to the extended PS00307 pattern)**.

We start by providing an in-depth study of grammars produced to describe the extended PS00307 pattern which include calcium and manganese binding sites. Through this analysis, we will use the 3D structure of a legume lectin protein, i.e. 1FAT, to visualise the structure of the site and its description as provided by the grammar parse trees. We will focus our attention on grammars based on residue accessibility, calcium propensity and manganese propensity, since these grammars have been shown as being the most informative to describe the PS00307 pattern. Figure 2a) shows the 3D structure of the extended PS00307 pattern. Figure 2b) displays Hydrogen-bonds involved in ligand binding and beta sheet which exhibit anti-parallel β-sheet type relationships.

The grammar generated for this pattern based on accessibility is composed of the following rules associated with their normalized probability. p, z and n express respectively high, average and low accessibility.

A → BC rules:

S → TS (0.49) | Vp (0.51)

T → XU (1.00)

U → nz (0.49) | zz (0.51)

V → pn (0.53) | pz (0.47)

W → zp (0.15) | TU (0.85)

X → VW (1.00)

A → a rules:

    A/L R/K N/M D/F C/P Q/S E/T G/W H/Y I/V

n: 0.01 0.10 0.11 0.11 0.00 0.10 0.14 0.04 0.00 0.00

   0.00 0.18 0.00 0.00 0.05 0.10 0.07 0.00 0.00 0.00

z: 0.11 0.05 0.05 0.04 0.01 0.05 0.02 0.09 0.10 0.03

   0.03 0.00 0.05 0.00 0.08 0.05 0.07 0.03 0.09 0.05

p: 0.00 0.00 0.00 0.00 0.15 0.00 0.00 0.00 0.03 0.14

   0.13 0.00 0.11 0.18 0.00 0.00 0.00 0.13 0.03 0.11

**Figure 3:**
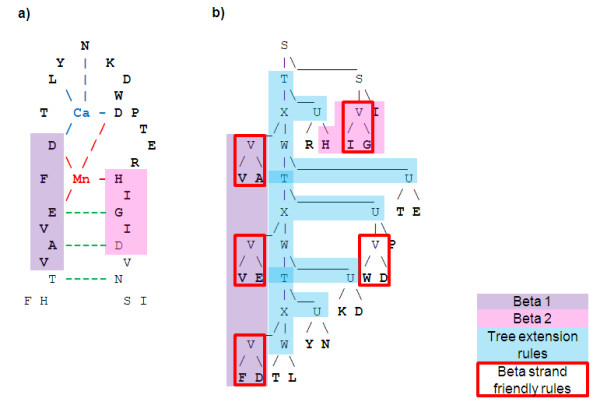
**a) Residues involved in the two beta strands b) Parse tree of accessibility based grammar**.

As shown on the grammar parse tree, see Figure 3b), there is a set of context free rules T→XU, X→VW, W→TU which is repeated to elongate the tree. Moreover, the derivation of X→VW is V→pn|pz. Since all amino acids which show high accessibility propensity, i.e. L, M, F, C, W, I and V, have also high beta sheet propensity [64], the V rule is beta strand friendly. Therefore, this grammar imposes a constraint between the length of the loop and the first beta strand, see Figure 3a).

Similarly, the rule S → Vp associated with V → pn|pz is Right-Hand Side strand friendly, which defines the second beta sheet, see Figure 3a) &3b).

Whereas the accessibility based grammar describes in particular the beta sheet which is present in the pattern, the magnesium propensity based grammar deals with magnesium binding. Rules of the 'A → BC' type for this grammar are the following:

S → Vn (0.43) | Vz (0.57)

T → zU (1.00)

U → Xn (0.26) | Tz (0.52) | TW (0.22)

V → nS (0.28) | XW (0.30) | VU (0.42)

W → np (0.67) | zp (0.31) | XW (0.02)

X → zW (1.00)

where p, z and n express respectively high, average and low magnesium binding propensity.

**Figure 4:**
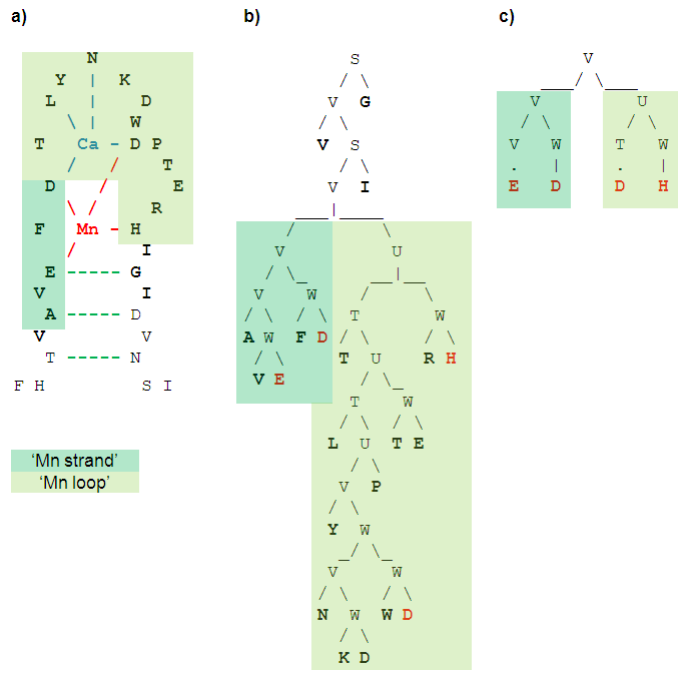
**a) Magnesium binding site divided in the strand and loop parts b) Parse tree of magnesium propensity based grammar c) Nested loop expressing relationships between residues interacting with magnesium**.

The parse tree of this grammar reveals the magnesium binding site is divided between strand and loop parts at V → VU branching rule, see Figure 4a), 4b) &4c). The derivations of V on the strand side and U on the loop side impose the presence of W rules. Since any W rule derivation exits with W → np|zp, which is magnesium binding friendly, both sides of the magnesium binding sites must include magnesium binding residues.

Finally, the calcium propensity based grammar defines not only the calcium binding part of the pattern, but more generally the pattern's structure. 'A → BC' type grammar rules are the following:

S → TW (0.50) | XS (0.50)

T → TW (0.42) | XW (0.58)

U → zp (1.00)

V → VU (0.06) | zz (0.94)

W → VW (0.46) | Vz (0.21) | pn (0.32)

X → VU (0.64) | XU (0.36)

where p, z and n express respectively high, average and low calcium binding propensity.

**Figure 5:**
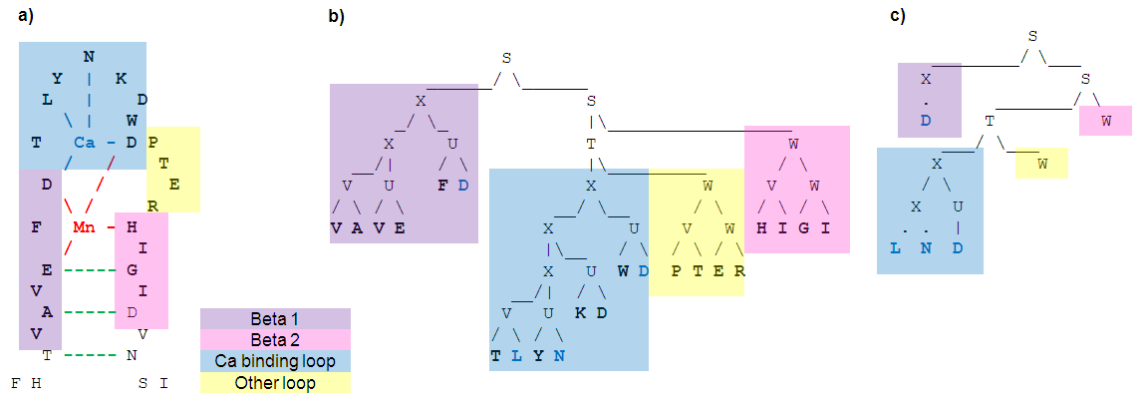
**a) Site divided in two strand and two loop parts b) Parse tree of calcium propensity based grammar**.

The parse tree of this grammar decomposes neatly the site in four parts: Beta1, Ca binding loop, Beta2 and rest of the loop, see Figure 5a), 5b) &5c).

**Figure 6:**
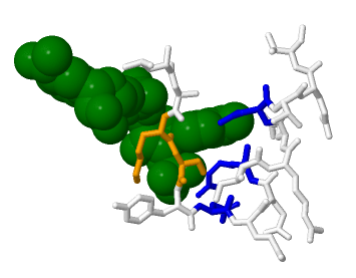
**3D structure of the PS00063 pattern in 1MRQ where the ligand (NAP) is shown in green, binding residues from the rule chain A in orange and binding residues from the rule chain B in blue**.

The second way of reading grammars, namely analysis of highly probable production chains and especially cycles, is demonstrated on the NAP binding pattern PS00063 which is found in some aldo- and ketoreductases. The protein structure of 1MRQ is used for illustration (see Figure 6 for a 3D stick model of the binding site).

Rules of the 'A → BC' type for the NAP propensity grammar for this pattern are the following:

S → zU (0.81) | Sz (0.19)

T → pV (0.72) | Tz (0.28)

U → zT (0.72) | Uz (0.28)

V → pU (0.24) | Vz (0.23) | Sp (0.19) |

V → zp (0.23) | np (0.11)

where p, z and n express respectively high, average and low NAP propensity.

For this grammar, there exist two cyclic rule chains of high probability (excluding recursive rules such as T→Tz) which start from non-terminal T.



Cycle A defines a NAP binding fragment consisting of two consecutive amino acids of high NAP propensity. This corresponds to the well conserved LYS270 and SER271 of 1MRQ, which are seen in orange in Figure 6.

Since the termination of any derivation of this grammar imposes either V→np or V→zp rules and another T→pV production (the only non recursive T rule) is required to end the cycle B, the shortest parse tree terminating cycle B (see above) would display the following pattern '**p**zz**p**yp**p**', where y represents either z or n. Residues of high NAP propensity show a '*i *→ *i*+3' periodicity which could suggest the presence of a NAP binding site involving an alpha helix. The derivation tree of 1MRQ shows that compared to the shortest predicted parse tree, the recursive rule T→Tz is used to extend the original pattern to '**p**zz**p**zp*z***p**' (*i*→*i*+3/*i*+4) which corresponds to the substring '**N**EQ**R**IRQ**N**'. Analysis of the PDB model confirms this pattern defines a helix. Moreover, ASN273, ARG276 and ASN280, which are seen in blue in Figure 6, are indeed very close to the ligand and provide an NAP binding environment. Ligplot data [59] show that ARG276, and ASN280 are actually involved in the bound state of the molecule.

**Figure 7:**
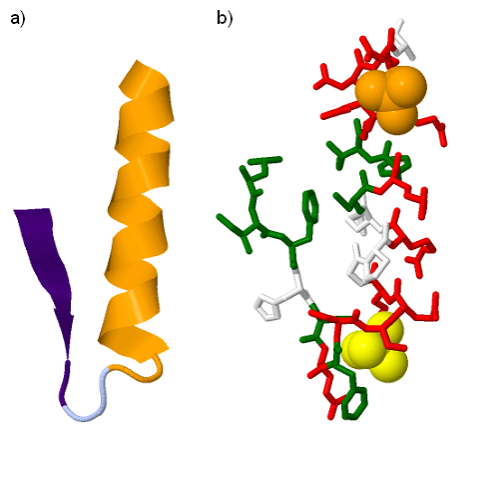
**3D structure of the PS50206 pattern in 1CWS: a) cartoon model coloured according to the parse tree of the accessibility based grammar (see Figure 8a); b) stick model including SO4 (orange) and WO4 (yellow) ligands**. Residues shown in red, grey and green colours have respectively high, average and low accessibility.

Finally, we analyse the SO4 binding site associated to PS50206 profile of m-phase inducer (MPI) phosphatase using both parse trees and grammar rules. This region is illustrated using cartoon and stick 3D models of 1CWS (see Figure 7).

**Figure 8:**
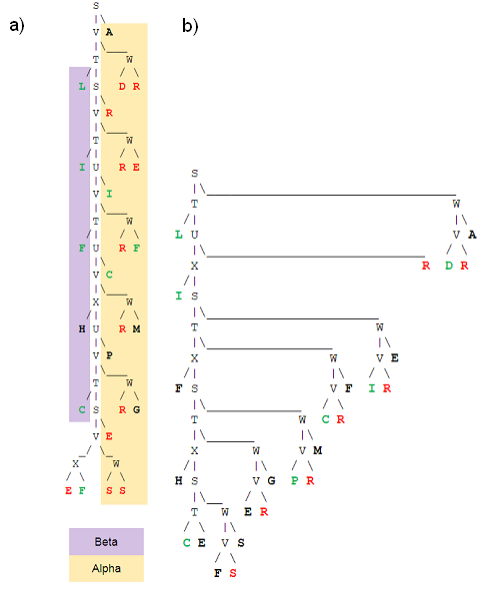
**Parse trees of a) accessibility and b) SO4 propensity based grammars for PS50206 pattern in 1CWS**. Red, black and green colours express respectively high, average and low level of the property of interest.

The parse tree of the accessibility based grammar for the region is shown in Figure 8a). This tree shows a strong asymmetry with a mainly hydrophilic left side and a hydrophobic right side. This suggests very different structural properties between these parts of binding site. Figure 7a) reveals a beta-sheet on the left and an alpha-helix plus one disturbed but clear turn on the right. In addition to these features which could have been obtained using standard secondary structure prediction methods, the parse tree also highlights the creation of a hydrophilic environment between the right side hydrophilic amino acids close to the tree root (i.e. I and C) and the left side amino acids. This is confirmed in Figure 7b) where the side chains of right hand side CYS484 and ILE487 are directed towards the beta sheet.

Using the parse tree of the SO4 propensity based grammar (Figure 8b), some insight can be provided regarding SO4 binding. Contrarily to the hydrophilic side of the site which is composed of residues showing low SO4 propensity, the hydrophobic side appears as a good candidate for SO4 binding. More specific information can be obtained through the analysis of the SO4 propensity based grammar whose rules of type 'A → BC' are as follows:

S → TW (1.00)

T → XW (0.51) | nU (0.25) | nz (0.25)

U → Xz (0.39) | Xn (0.34) | Xp (0.27)

V → np (0.57) | zp (0.36) | nz (0.07)

W → Vz (0.94) | Vp (0.06)

X → zS (0.54) | nS (0.46)

where p, z and n express respectively high, average and low SO4 binding propensity.

Two most probably cyclic chains are as follows:



where the most likely derivation of W is: W → Vz and V → n|z p

From cycle A, the following pattern is revealed: 'ySy**p**zy**p**z', where y represents either z or n. Since S can be substituted by the pattern itself, after a substitution the patterns become 'yySy**p**zy**p**zy**p**zy**p**z'. This shows a '*i*→*i*+3' periodicity of SO4-friendly residues which is consistent with the presence of an alpha-helix. In addition, the derivation of the less likely cycle B produces the following pattern: 'nySxy**p**z' where x represents any SO4 propensity. Then, if S is substituted by the pattern coded in cycle A, the new pattern models a '*i*→*i*+3/*i*+4' periodicity, 'nyySy**p**zy**p**zxy**p**z', which is also typical for helices.

This result combined with the low SO4 propensity of the hydrophobic side of the binding site suggests that SO4 binding would involve the arginine-rich ridge of a helix. This is confirmed by Ligplot which shows that ARG488 and ARG492 are involved in SO4 binding.

This analysis of grammars describing ligand binding sites has shown that probabilistic context-free grammars allow the production of binding site descriptors which are human-readable and, hence, provide some insight into biologically meaningful features. Moreover, each of these grammars relies on high probability rules which could not be expressed with regular grammars. Therefore, this confirms that the description of many ligand binding sites benefits from the expressive power of context free grammars.

### Stochastic Context-Free Grammars for sequence annotation and binding site detection

In order to demonstrate that, not only SCFG based descriptors are meaningful, but are powerful at both annotating sequences and detecting binding sites, we first evaluate them on sites which can be expressed quite successfully by a PROSITE pattern. In this section, all results are produced using grammars containing a full set of rules.

Since each SCFG deals with one amino acid property at a time, scores obtained by several grammars need to be combined to obtain optimal results (see Methods for details). Table 2 shows TP and TN rates for grammars based on different properties, their combinations and their comparison with scores obtained by PROSITE patterns. This short pattern - only 12 residues - is the anion exchanger pattern (PS00219). The table reveals that charge and van der Waals volume are important features for the expression of this binding site. Moreover, SCFGs allows detecting relevant sequences missed by the PROSITE pattern.

**Table 2:** TP and TN rates for sequence annotation by grammars obtained for PS00219 pattern

	**Charge**	**Van der Waals volume**	**Beta sheet propensity**	**Charge + volume**	**PROSITE accuracy**
**TP rate**	0.79	1.00	0.62	1.00	0.67

**TN rate**	0.96	0.97	0.42	0.99	1.00

Since each SCFG deals with one amino acid property at a time, scores obtained by several grammars need to be combined to obtain optimal results (see Methods for details). Table 2 shows TP and TN rates for grammars based on different properties, their combinations and their comparison with scores obtained by PROSITE patterns. This short pattern - only 12 residues - is the anion exchanger pattern (PS00219). The table reveals that charge and van der Waals volume are important features for the expression of this binding site. Moreover, SCFGs allows detecting relevant sequences missed by the PROSITE pattern.

**Figure 9:**
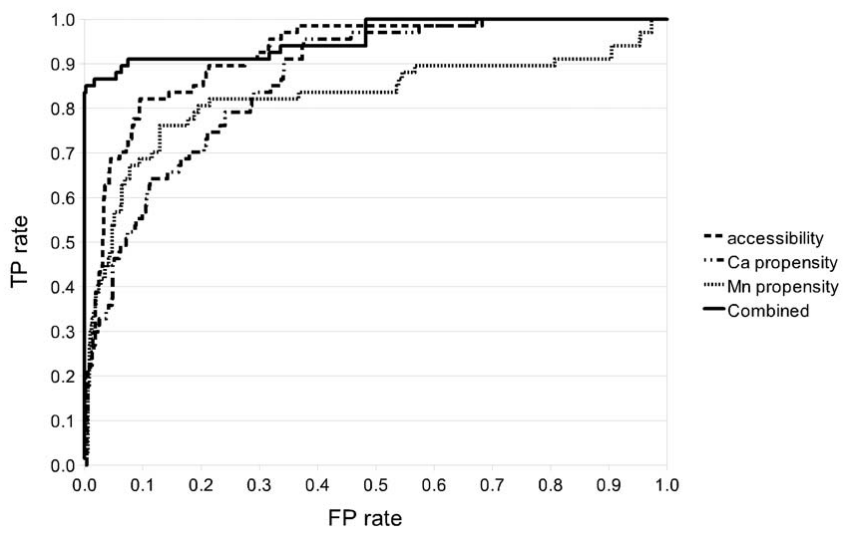
**Annotation ROC curves for PS00307 single property and combined grammars**.

**Figure 10:**
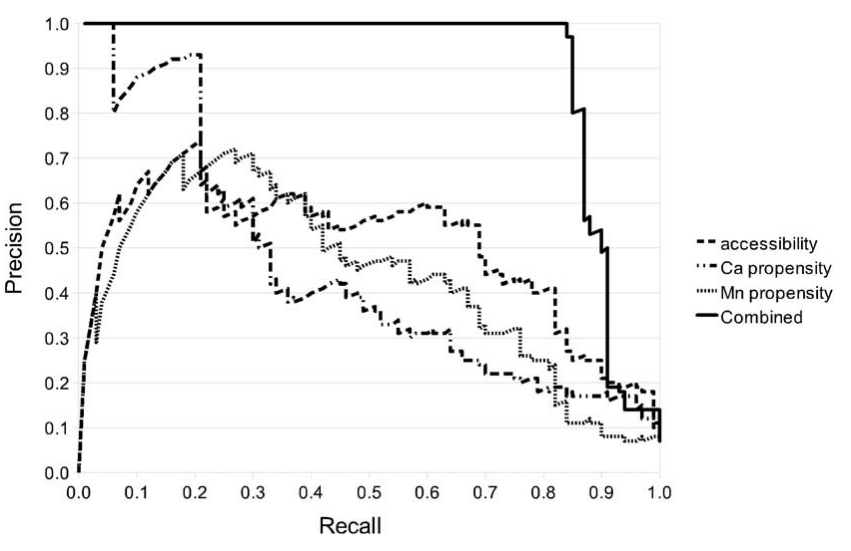
**Annotation RPC curves for PS00307 single property and combined grammars**.

A more complete performance analysis of different grammars (all constrained SCFGs) is provided in Table 3 for PS00307 in terms of Precision, Recall and maximum F1 is shown. We also provide in Figure 9 the Receiver Operating Characteristic (ROC; Egan, 1975). Since negative-to-positive ratios in our datasets are quite high (between 6 and 13), ROC curves may present an optimistic assessment of the performances of our framework. Therefore, we also show in Figure 10 a Recall-Precision Curve (RPC) [60] which has been proposed as a better alternative [61]. Although accessibility and, Ca and Mn propensity are key properties of the residues involved in this binding site, they need to be combined to produce good results.

**Table 3:** Sequence annotation by grammars obtained for PS00307 pattern

	**Max F1**	**Precision**	**Recall**	**Rc|Pr = 1.0**
**Accessibility**	0.61	0.59	0.63	0.00

**Ca**	0.44	0.43	0.45	0.06

**Mn**	0.52	0.47	0.57	0.00

**Combination**	0.91	1.00	0.84	0.84

Results obtained for PS00219 pattern by using SCFGs are near perfect. Pattern PS00307 appeared to be more difficult, but Recall for 100%Precision is still very high 0.84. The results for combined grammars are also very good for PS00063: Recall of 0.81 for 0.93 Precision and maximum F1 of 0.87 (see Table 4). Only 69% of sequences in the positive set are recognised without FP. This slightly worse result can be explained by the fact that unlike the two other patterns, the pattern covers only a part of the binding site to relatively huge NAP molecule. Therefore many key dependencies were not available to the grammars.

**Table 4:** Sequence annotation by combined grammars obtained for PS00063 pattern

**Max F1**	**Precision**	**Recall**	**Rc|Pr = 1.0**
0.87	0.93	0.81	0.69

Since correct annotation does not imply correct detection, both tests - annotation and detection - are necessary to prove the functionality of the approach. In order to evaluate capabilities of detection, a number of tests were carried out. In Table 5 results for PS00307 for the combined grammar most successful in annotation task are shown.

**Table 5:** Binding site detection by grammars obtained for PS00307 pattern.

	**Detection (Exact)**	**Detection (50% coverage)**
**Accessibility**	0.76	0.99

**Ca**	0.45	0.85

**Mn**	0.37	0.88

**Combination**	0.75	1.00

Detection (Exact): the peak is expected to be exactly at the position of the pattern; Detection (50% coverage): the peak is expected to be no longer distance than 50% of the pattern length (here: 10)

As an outcome of this evaluation, performance of detection appears to be good. In the most difficult task, where the highest peak was demanded to be exactly at the position of the pattern, success rate was 75%. When 50% coverage, i.e. 10 residues displacement for PS00307, was allowed, success rate rose to 100%. Similar outcomes were obtained for the other patterns where detection results were in line with annotation results.

To conclude, our system managed to achieve good accuracy in both annotation and detection. The results confirmed suitability of our approach in integrating amino acid properties in our grammars and combining obtained grammars. It shows that these strategies together with appropriate choice of the properties relevant to the pattern provide satisfactory solutions to the requirement of alphabet size reduction.

The remaining part of this paper will only show annotation results since it makes comparisons with profile HMM performances easier.

### Constrained grammar evaluation

In this section we evaluate the approach consisting on constraining the initial grammar structure as described in the Methods section. It imposes a bias in the grammars so that they use context-free features and it allows increasing the number of non-terminal while keeping a manageable total number of rules (see Appendix D). Comparisons between performances obtained by SCFGs with a full set of rules - standard SCFG - and a constrained set - NestedNT SCFG - is provided in Table 6 where results regarding annotation task for PS00063, PS00307, MPI phosphatase and zinc finger meta-patterns are provided.

**Table 6:** Performance comparison of standard and NestedNT SCFGs

	**Max. F1 (Rc|Pr = 1.0)**							
	***PS00063***		***PS00307***		***MPI phosphatase***		***Zinc finger***	

	Standard	NestedNT	Standard	NestedNT	Standard	NestedNT	Standard	NestedNT

**Acc**	0.31	0.36	0.16	0.61	0.49	0.76	0.25	-

**NAP**	0.18	0.18	-	-	-	-	-	-

**Ca**	-	-	0.30	0.44	-	-	-	-

**Mn**	-	-	0.46	0.52	-	-	-	-

**SO4**	-	-	-	-	0.65	0.79	-	-

**Zn**	-	-	-	-	-	-	0.78	0.81

**Comb**	0.38	0.53	0.72	0.91	0.75	0.89	0.61	-

NestedNT SCFGs performed consistently better than standard SCFGs, especially concerning Recall for 100%Precision. These results suggest that increasing the number of non-terminals allows improving performance by increasing expressive capabilities. Analysis of parse trees shows that more than 6 independent NTs would be required to cover all important structural features. Moreover, examination of grammar structures produced with different parameters confirmed that constrained grammars were more consistent in their structure than standard SCFGs.

### Performance comparison of SCFGs with profile HMMs

Since we have already demonstrated that unlike HMM profiles, rules of SCFG are human readable and can be used to gain some biological insight about binding sites, in this section comparison between the two techniques is limited to annotation results.

As PS00219, PS00063 and PS00307 patterns were optimised for PROSITE, this method has an intrinsic advantage compared to Profile HMMs and SCFGs for these patterns in this experiment. Moreover, since MPI phosphatases are a subset of Rhodanese-like proteins which can be expressed by a domain profile (PS50206), it is expected that profile HMMs would perform well in annotating this family. Since PROSITE scores are calculated using the whole Swiss-Prot/TrEMBL database, comparison with other methods based on Precision and therefore F1 statistics may not be fair. Therefore, we use Recall for 100%Precision of Profile HMMs and PCFGs, and PROSITE Recall to evaluate PROSITE performance against the others. Table 7 shows comparison of results between the methods for our patterns of interest.

**Table 7:** Performance comparison of PROSITE patterns, Profile HMMs and SCFGs

	**Size**	**Method**	**Max F1**	**Pr**	**Rc**	**Rc| Pr = 1.0**
**PS00219**	12	PROSITE	0.94	1.00	0.89	0.89
	12	SCFG	1.00	1.00	1.00	1.00

**PS00063**	16	PROSITE	0.81	1.00	0.81	0.81
	16	Profile HMM	0.95	1.00	0.91	0.91
	16	SCFG	0.71	0.79	0.65	0.29

**PS00307**	7	PROSITE	0.52	0.36	0.94	0.94
	25	Profile HMM	1.00	1.00	1.00	1.00
	25	SCFG	0.91	1.00	0.84	0.84

**MPI phosphatase**	-	PROSITE	No PROSITE pattern			
	25	Profile HMM	0.96	1.00	0.92	0.92
	25	SCFG	0.94	0.98	0.89	0.85

**Zinc finger**	10-26	PROSITE	Out of scope			
	26	Profile HMM	0.79	0.76	0.82	0.21
	26	SCFG	0.81	0.75	0.87	0.21

**Figure 11:**
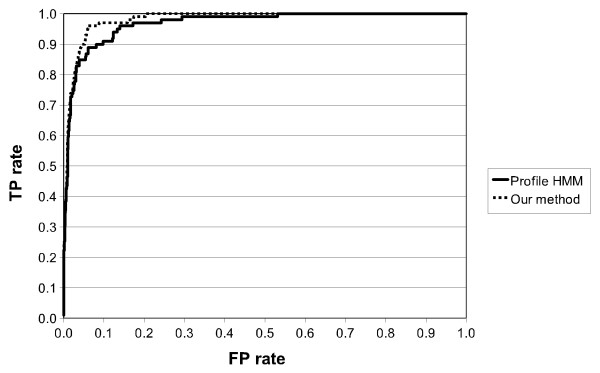
**ROC curves for a Profile HMM and a single Zinc propensity grammar**.

**Figure 12:**
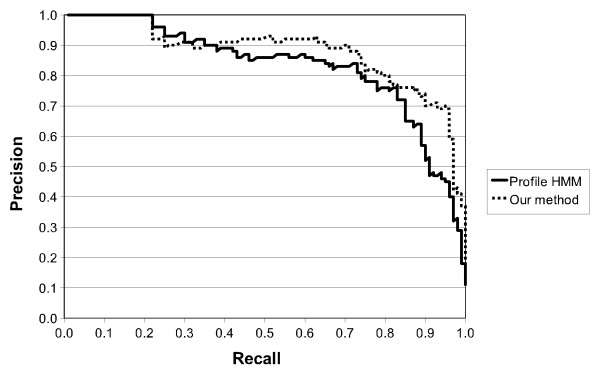
**RPC curves for a Profile HMM and a single Zinc propensity grammar**.

As expected, although SCFGs scores are generally good, PROSITE and Profile HMMs outperform SCFGs when dealing with standard PROSITE patterns. PS00219 is an exception where SCFGs obtain perfect score. Results achieved by Profile HMM and SCFG for MPI phosphatase pattern are very similar, with the former only slightly superior over the latter. In the case of the zinc finger meta-pattern, Table 7 does not reveal any significant difference between performances of Profile HMMs and SCFGs. These results validate our assumption that SCFGs gain efficiency from higher expressiveness and, despite operating on a reduced protein alphabet, can be at least as efficient as lower-level grammars, i.e. PROSITE patterns and Profile HMMs, built on sequences of amino acid identities. To investigate further the meta-pattern results, ROC and RPC are provided in Figure 11 and 12.

These curves show that although our stochastic grammars are based here on a single feature - zinc propensity - they perform slightly better than Profile HMMs. Although none of the other tested properties allowed improving SCFGs results, we believe there is still some space for improvement if suitable properties could be combined to zinc propensity.

Study of false negatives produced by these two methods at their maximum performance level in the term of F1, shows 13 sequences were rejected by SCFG while 17 by Profile HMM. Since only 4 sequences belonged to both groups, this seems to confirm both schemes have very different definitions of this binding site which might be regarded as complementary.

## Discussion

This study demonstrates the capability of the framework we propose to take advantage of the expressive power of Stochastic Context Free Grammar for analysis of protein sequences. First, we have shown with a few examples that the analysis of sequence based SCFG rules allows gaining an insight into the 3D structure of binding sites. This is a unique feature of our approach compared to, for example, HMM based methods which only produce 'black box' descriptors. Our analysis of grammar structures has revealed that the ability of expressing branched and nested dependencies is essential to describe some binding sites which can be seen as displaying such relationships through the intermediate of a ligand. This confirms the theoretical potential of our method in describing sites which are out of the scope of HMM profiles. It is important to be aware that many sites, such as parallel β-sheets, display dependencies which are beyond the expressiveness of SCFGs and, therefore, are not suitable candidates for our approach.

Secondly, we have demonstrated that our SCFG based system can be practical and accurate to annotate proteins and detect binding site patterns. This confirms that the various strategies developed to deal with the challenge of using SCFGs for proteins sequences were appropriate. Our approach of integrating quantitative properties of amino acids into the SCFG framework has shown to be an efficient method to reduce the size of grammars. Moreover, properties based on ligand propensity were especially useful. In addition, combining grammars typically yielded in better scores than those obtained by any single property grammar. Furthermore, experimentations, where grammar structures are constrained, suggest that restricting the grammar induction search space using heuristics is an approach which merits further investigation. Results show that our approach usually does not perform as well as Profile HMMs in annotation task when dealing with binding sites which can be well expressed with rigid regular grammars. This can be explained by the fact our scheme relies on a reduced amount of information, i.e. amino acid properties instead of amino acid identities,

The main benefit of the system we propose is that - when considering only rules with significant probabilities - both structure and rule probabilities of context-free grammars are learned automatically without introducing constraints specific to the targeted sites. Such grammars of relatively simple structure are human-readable; hence they could become valuable sources of information for molecular biologists. Another advantage of our framework is that, unlike other methods, it does not rely on sequence alignments. Therefore, as encouraging results with zinc finger meta-pattern suggest, our system may be particularly suited to deal with patterns shared by non-homologous proteins.

## Conclusion

We have presented a novel Stochastic Context-Free Grammars based framework relying on quantitative representation of amino acid properties. The SCFG based system for protein sequence analysis was tested on several data samples in various configurations. First, we have shown the produced binding site descriptors are human-readable and their analysis can provide biological insight into the structures of their associated binding sites. To our knowledge, no other type of binding site descriptors can reveal subtle interactions as described by SCFGs. Secondly, by achieving high Precision and Recall in annotation and very good detection rates our system proved to be a practical tool for protein pattern recognition. Moreover, results for the zinc finger meta-pattern which outperformed Profile HMMs suggest that meta-patterns are one of the fields where application of SCFGs can be especially useful. In addition, since both approaches produced different false negative, SCFGs can be seen as complementary to existing Profile HMM based methods. This suggests our SCFG framework could be used to improve those methods. This study also supports the idea that binding site regions which could be seen as involving indirect nested dependencies between residues are prime targets for our framework.

An increase of the number of non-terminal symbols allows representing a larger variety of relations within a binding site. Additional constraints to grammars enable the use of more symbols and facilitate the learning process. Indeed, our experiment showed that, generally, constrained grammars produced better results. For future work, we intend to further our research into constrained grammars by inferring optimal grammar structure. We will also implement secondary structure based grammars to take advantage of this higher level property. Furthermore, our procedure of grammar combination will be refined by customising each grammar's weight to reflect the entropy of underlying properties in the training samples and single grammar performance. Finally, we plan to develop a web tool allowing interactive analysis of binding sites encoded by SCFG based descriptors.

## Methods

### Principles

We present a grammar based system for analysis of protein sequences. The complexity of amino acid interactions led us to adopt a Context Free Grammar framework. Moreover, the difficulty of producing negative samples guided our selection towards a stochastic scheme (a formal definition of stochastic context-free grammars is provided in Appendix A). Since in the context of protein sequences the structure of the grammar is originally unknown, it needs to be induced. In this work a genetic algorithm is used for this purpose (see Appendix C for details). The general principle behind our framework is to start the learning process from a set containing all possible rules and infer their probabilities. Although this approach leads to quite large sets of rules even for moderate alphabets, it avoids bias which can be introduced by additional constraints. Moreover, an inherent property of proper stochastic grammar is that the distribution of probabilities onto a small number of rules, which express well the pattern of interest, gives better scores than even distribution onto all possible rules. Consequently, a natural trend during grammar evolution is the reduction of probabilities of rules that are unnecessary. Therefore, after grammar induction, the final set of rules can be pruned to omit rules which will have only a limited impact on the overall score of a scanned sequence.

In principle, the number of context-free rules for a given number of non-terminal symbols can be infinite. However, by transforming a CFG into a Chomsky Normal Form (CNF, see Appendix A for details), the number of rules is bound by the cube of the number of non-terminals. Therefore, this formulation will be used in our framework. Another advantage of CNF is that it can be parsed by the efficient polynomial probabilistic CKY parser (see Appendix B).

Since a protein can be fully defined by a string composed of 20 different characters, protein grammar is expected to rely on a large set of terminals (20 amino-acids). Therefore, the space containing the possible rules needed to describe the protein language is enormous and cannot be searched in a finite time by current induction techniques. In order to deal with the size of the protein alphabet, we introduce quantitative properties of amino acids into our SCFG framework to reduce the number of symbols in a grammar (additional strategies used to handle computational complexity are detailed in Appendix D). For each given property, our method relies on defining all the terminal rules of the form A → a and associating them with proper probabilities. Three non-terminal symbols (Low, Medium and High) are created to represent low, medium and high level of the property of interest, e.g. small, medium or large van der Waals volume. These non-terminals are later called 'property non-terminals' or pNT. Those rules have therefore the following format:



where each amino acid (a_i_) is associated with each of these non-terminals with a probability (Pr).

For a given property, probabilities are calculated using the known quantitative values, *pval*, associated to the amino acids, a_i_, using the following equations:



These probabilities are then normalized so that they are proper, i.e. the following equation is true:



Since all terminal rules are fixed with given probabilities, only the probabilities of the subset of rules in the form of A → BC are subject to evolution. However, relations expressed by these rules only refer to the 3 property levels instead of the 20 amino acid types. Moreover, to avoid trivial solutions, non-terminals which are Left-Hand Side (LHS) symbols in the terminal rules are prohibited from being LHS non-terminal symbols of these other rules. The LHS non-terminals of A → BC are later called 'independent non-terminals' or iNT.

**Figure 13:**
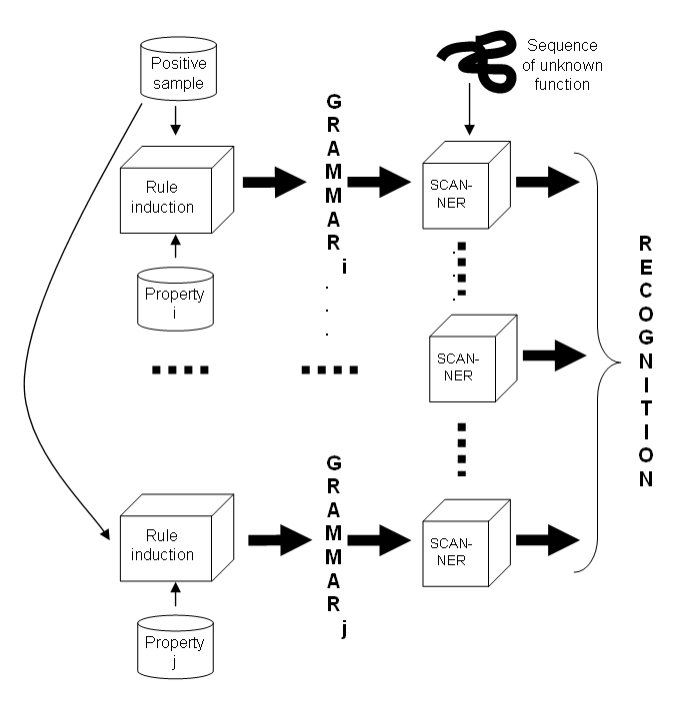
**General scheme of the method**.

A drawback of this significant reduction of the size of the possible rule space is that properties of each amino acid are represented by a single feature in the induced grammar. In our framework (see Figure 13), this limitation is overcome by generating one grammar per relevant physiochemical property. Protein sequences are parsed for each grammar and their parsing scores are combined to achieve more robust results: the final score is an arithmetic average of the scores obtained for each single property grammar.

### Choice of amino acid properties

Our method relies on the selection of amino acid properties in order to deal with the size of the protein alphabet by defining specific terminal rules based on their quantitative values. These values are collected from the AAindex database which provides quantitative estimates of the 20 amino acids for over 500 properties [43-45]. They are clustered into 6 categories which can be broadly labelled as beta propensity, alpha and turn propensities, composition, physiochemical properties, hydrophobicity and others. Representatives of the well defined properties, i.e. the 5 first ones, are selected by choosing properties which are the closest to the centres of the property clusters defined in the AAindex database. They are defined using their AAindex property codes as:

• Average relative frequency of beta-sheet, KANM800102 [46],

• Information measure for middle helix, ROBB760103 [47],

• Relative frequency of occurrence JOND920101 [48],

• Normalized van der Waals volume, FAUJ880103 [49],

• Information value for accessibility with average fraction of 35% (called later "accessibility"), BIOV880101 [50].

In addition to these properties, we also used the net charge of amino acids. Values are obtained by combining net, positive and negative charge indices from KLEP840101 [51], FAUJ880111 and FAUJ880112 [49] respectively. Moreover, since we target protein binding sites, ligand binding propensities of amino acids can also be very informative. Those propensities are calculated by standardising the ligand binding statistics provided by the Molecular Structure Database of EMBL-EBI [52].

Although in principle, grammars could be generated for all of those properties, computational constraints make it impractical. Therefore, an appropriate set of properties is chosen using expert knowledge for each binding site of interest.

### Constrained grammars

An alternative to inferring a grammar with a complete set of rules is to produce a grammar where constraints are introduced in the design of an initial rule set. This brings a couple of advantages. First, for a given number of rules the number of non-terminals (NT) can be increased. Secondly, it allows imposing a bias in the grammars so that they use context-free features (i.e. nesting and branching) instead of regular features which may lead to local minima in the rule inference search space. The rationale behind this concept is that it requires at least two rules in Chomsky Normal Form that collaborate with each other to represent nested dependencies. As evolution of probabilities of rules with different LHS non-terminals is independent, the bias towards nested solutions may need to be enforced in the grammar structure. To achieve this, we propose to constrain rules of the type A → BC.

To describe these constraints, we shall recall the following definitions:

• Non-terminal symbol which represents explicitly an amino acid property is called 'property NT' or pNT.

• Non-terminal not representing explicitly an amino acid property is called 'independent NT' or iNT.

In the full rule set, A must be iNT while B and C can be any NT:



Therefore, for 7 NTs including 3 pNT this results in a total of 196 rules.

Our constraints, called Nested NT, aim at imposing to the grammar the production of nested relations, while the structure of the grammar is kept general. This is performed by dividing independent NTs into two equal and mutually exclusive subsets. They are named 'odd independent NT' or oiNT, and 'even independent NT' or eiNT. Then restrictions on the rule set are introduced: they are represented by the following 3 subtypes of rules:



The generalised nested relations are produced by the first and second rules which impose switching in the course of derivation between oiNT and eiNT. Additionally, the third rule creates a branch if the NT of the free form is iNT.

This scheme produces only 76 rules for 7 NT. Hence, for NestedNT the number of NTs can be increased from 7 to 9 while keeping the number of rules manageable (i.e. 162 rules, more details in Appendix D).

### Evaluation and datasets

The large size of the amino acid alphabet, the non-trivial character of dependencies between residues and the high computational complexity of Context-Free Grammars make the task of learning efficient SCFGs for protein pattern detection very challenging. In order to assess the performance of our framework, grammars evolved under our scheme were first utilised to gain insight into binding site biological features. Then, they were evaluated in their ability to detect the precise positions of a binding site within a sequence and to annotate protein sequences. Finally, results were compared to those obtained from Profile HMMs. These profiles were built using the latest version (v 2.3.2 at the time of writing) of the standard HMMER package [20] where standard parameters were applied and same positive training data sets as SCFGs were used for training.

In the annotation task, the parser returns either the presence or the absence of the binding site of interest. To evaluate our results, we use the F1 measure, a harmonic mean of Precision and Recall, defined as follows:



Since F1 is more conservative measure than arithmetic or geometric average, it allows finding the optimal threshold to distinguish between presence and absence. Typically, the highest score generated by a properly induced grammar occurs in the position of the binding site. However, precise site detection is not a requirement for correct annotation.

Binding site patterns used to evaluate our framework are based on PROSITE entries, since PROSITE is the database containing the most comprehensive library of protein patterns; many of them describe binding sites [56]. While PROSITE contain both regular expression based patterns and domain profiles, in this document when we mention PROSITE patterns we refer to the former. Since PROSITE patterns are regular expressions, there is some bias towards representing binding sites with strong regular features. However, some PROSITE patterns are associated with relatively high false positive and false negative rates and therefore are suitable for testing our scheme. Moreover, many PROSITE patterns can be associated to a single binding site environment, e.g. 11 patterns are linked to zinc fingers. Since our framework does not rely on the alignment of protein sequences, which implies some homology, it makes it possible to combine patterns functionally similar but associated with different protein families. Those meta-patterns can be particularly powerful in detecting binding sites in sequences without homologues.

Three PROSITE binding site patterns of various sizes and complexity were selected to show that our general approach is valid. Then, using a PROSITE domain profile, we analysed a subset of the proteins it describes to compare our method with a HMM profile. Finally, a zinc finger meta-pattern that we created using 7 zinc finger PROSITE patterns is processed to demonstrate the performance of our framework on binding sites which belong to different protein families. For each pattern of interest, positive and tests sets were based on the associated PROSITE dataset - no negative training set is required in the learning process. Ideally the negative test set should have been defined as a representative set of all protein sequences available in UniProt [57] which does not contain the pattern of study. Due to computational constraints a smaller set had to be defined. Therefore, the Protein Data Bank (PDB) [58] instead of UniProt was chosen as the database of reference.

## Authors' contributions

WD and JCN designed the methodology. WD implemented the methodology and processed data sets. WD and JCN performed data analysis. All authors contributed to draft the manuscript. All authors read and approved the final manuscript.

## Appendices

### A. Formal definition of stochastic context-free grammars

The formal definition of a context-free grammar G is the following [28]:



where V is a finite set of non-terminal symbols, T is a finite set of terminal symbols, P is a finite set of production rules and S is a special start symbol (S ∈ V). The sets V and T are mutually exclusive. Each rule from the set P has the following form:



where A ∈ V and X ∈ (V ∪ T)*. A context-free grammar may include rules with an empty Right-Hand Side (RHS) denoted as:



A given grammar is called λ-free if and only if there is no rule in the set P where a RHS symbol X is empty (λ). It has been proven that for each context-free grammar G, a λ-free grammar G' may be constructed:



where L(G) denotes the language generated by the grammar G. For each λ-free grammar G' = < V', T', P', S' >, one can find a grammar G" = < V", T', P", S'> that is equivalent to G' and is in the Chomsky Normal Form (CNF), in other words, its set P" consists of productions of two types:



where upper case letters refer to non-terminals and lower case letters refer to terminal symbols. Although the representation of grammars in Chomsky Normal Form is not the most compact one, context-free grammars in the CNF have a simple and well-defined form of rules. Therefore, this formalism is commonly used in computational linguistics and many efficient algorithms have been specially designed for it.

The definition of a Stochastic Context Free Grammar (SCFG) is similar to the definition of a non-probabilistic CFG, where probabilities are attributed to each rule:



Usually, probabilities of all productions for one Left-Hand Side (LHS) symbol sum to one; the SCFG is then called proper.

### B. Standardised parsing method for stochastic context-free grammars

The Cocke-Kasami-Younger (CKY) algorithm (designed in the 1960s) is one of the most common parsing methods for CFGs. It has a simple structure and is efficient: its computational complexity is polynomial O(n^3^) regarding string length. This is a bottom-up parser, i.e. the derivation tree is built starting from terminal symbols. It requires a context-free grammar in the Chomsky Normal Form, so that the string which is analysed gets shorter at every iteration step. In this work, we implemented a modified version of the CKY algorithm, called Stochastic CKY or SCKY [29], so that Stochastic Context Free Grammars can be parsed. The outcome of this procedure is the probability that a given sequence was generated by a certain grammar G (i.e. the sequence belongs to the language L(G)) instead of a Boolean value as in the case of non-probabilistic CKY. This probability is defined as a product of probabilities of all grammar rules involved in the construction of the corresponding parse tree. More formally, it can be described as follows [30]:

Given an input sequence W of N terminal symbols: W = a_1_, a_2_, ..., a_N_. Grammar G consists of R non-terminal symbols: α_1_, α_2_, ..., α_R _and a set P of L_T _terminal (p_1_: α_r _→ a_i_, Pr(p_l_)) and L_NT _non-terminal (p_m_: α_r _→ α_s _α_t_, Pr(p_m_)) rules. A table T of dimensionality N × N × R is constructed and initially filled with zeros. Then the following algorithm is applied:

   For each element *a*_*i *_of the input sequence *W *(i.e. for *i *= *1 *to *N*)

   for each terminal rule in the *P *set (i.e. *l *= *1 *to *L*_*T*_)

         if there is a production rule *p*_*l*_: α_*r *_→ a_i_,

                  the value of *T *at position [*i, 1*, α_*r*_] is increased by Pr(*p*_*l*_)

   Then, for *i *= *2 *to *N*

      and for *j *= *1 *to *N-i+1*

         and for *k *= *1 *to *i-1*

            for each non-terminal rule in the *P *set (i.e.*m = 1 *to *L*_*NT*_)

            if there is a production rule *p*_*m*_: α_*r *_→ α_*s *_α_*t *_in G,

                  the value of *T *at position [*i, j*, α_*r*_] is increased:

                  *T *[*i, j*, α_*r*_] + = *T *[*j, k*, α_*s*_] × *T *[*j *+ *k*, *i*-*k*, α_*t*_] × Pr(*p*_*m*_)

The value of T [i, j, α_r_] is consequently the probability that a sequence of terminal symbols:



(or the subsequence of the input sequence W between positions i and i+j-1) was derived from the non-terminal symbol α_r _of the grammar G. Therefore, after all steps, the value of T [1, N, 1] is the probability that the input sequence W belongs to the language L(G). This version of the algorithm, where a probability for a certain node is calculated as a sum of probabilities of all sub trees is called the Baum-Welch style SCKY algorithm [31].

If a probability for a certain node is calculated as a maximal probability instead of the sum from all sub trees:



one obtains a Viterbi style SCKY algorithm [32]. In this case the value of T [i, j, α_r_] is the probability that a sequence of terminal symbols a_i_, a_i+1_, ..., a_i+j-1 _(or the subsequence of the input sequence W between positions i and i+j-1) was derived from the non-terminal symbol α_r _of the grammar G in the Viterbi or most likely parse. T [1, N, 1] is therefore the probability that the input sequence W was generated by the grammar G in the Viterbi parse.

**Figure 14:**
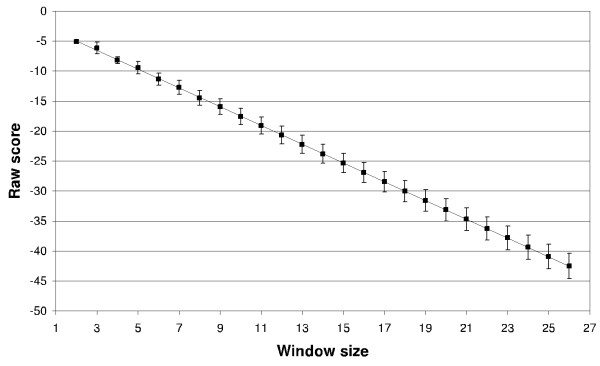
**Linear character of the change in average raw scores with increasing parse tree span**. Standard deviation is shown. Marked area denotes parse tree spans which cover at least one whole pattern instance from the learning set (pattern based on PS00307, see Datasets section for details).



In order to assign comparable scores to derivation trees of different spans, a scheme that allows for detection of the most probable node in the parsing tree was developed. The general idea is based on the fact that each additional level of parsing causes a certain decrease in the resulting probability. Therefore, the analysis of that effect can be used for standardisation based on a scaling factor which would allow comparing scores at different parse tree spans. A general approach to scaling within Viterbi-style CKY parsing scheme is to calculate normalized scores (Score') as follows:



where A compensates the decrease in score caused by linking another terminal (A → a), R compensates the decrease in score caused by invoking a rule (A → BC) and i is the parsing level. As it is difficult to calculate the values of A and R, an empirical scaling factor, F = RA, was introduced. It is based on the change in average raw (not scaled) scores with increasing parse tree spans calculated over a large negative dataset for a given grammar. Analyses performed for various grammars confirmed the constant character of this change across derivation tree spans (see Figure 14). This justifies the utilisation of one scaling factor per grammar, which is calculated as

where a is the coefficient of linear regression if the logarithm of the score is used instead of its raw value.

Standard deviation is shown. Marked area denotes parse tree spans which cover at least one whole pattern instance from the learning set (pattern based on PS00307, see Datasets section for details).

In our framework, Baum-Welch style is utilised in training and Viterbi style in scanning mode. Baum-Welch parsing was chosen for grammar induction to avoid rapid convergence to trivial local minima in the absence of a negative training set. On the other hand, according to our experiments Viterbi algorithm produces better discrimination between positive and negative samples and therefore it is more appropriate for scanning. Moreover, Sakakibara's work suggests that when a grammar is correctly induced, the most likely parse tree of a binding site sequence reflects its structural features [22]. In both cases, the basic version of the parser returns a score which is the log of probability rather than the probability itself. Therefore, it is the average log of probabilities for all training samples which is optimised in the training step.

### C. Learning method for stochastic context-free grammars

Since grammar rules are generally unknown in real world applications, efficient and robust methods are required to infer Stochastic Context-Free Grammars. There are two main approaches for learning grammars: Maximum A Posteriori (MAP) Expectation-Maximisation algorithms (EM) and evolutionary methods (Genetic Algorithms (GA) [33,34] or Genetic Programming (GP) [35]). The former was employed for SCFGs of known structure (only probabilities were trained) [22,23]. Moreover, a MAP estimation process was designed based on single base and base pair (local neighbours) frequencies, mutation rates and, finally, rule probabilities [37]. Among evolutionary approaches, GP was successfully applied to derive grammars for small domain-specific non-probabilistic Context-Free languages using positive and negative examples [38]. A tabular representation for CFGs was also proposed to reduce the problem of learning CFGs to the problem of merging and splitting non-terminals according to positive and negative samples [39]. Furthermore it was shown that it is possible to learn grammar from positive examples only, if precise information of the grammatical structure of the unknown grammar is available [27]. Finally, steady-state distributed GA was applied for a version of SCFGs called Biased Weighted Grammars [40]. In this approach, grammar rules were divided into two parts: a real and constant 'bias', and an integer 'weight' which was evolved. Following experiments on theoretical grammars and short sequences, the authors reported faster convergence and a better success rate and grammar compactness than grammars obtained by the Inside Outside version of the EM algorithm.

Following successful applications of evolutionary algorithms to SCFG [39,40] and our previous experience in CFG learning [41], we chose a Genetic Algorithm for grammar induction. In the design of the GA we adopted the approach where a single individual represents a whole grammar. This strategy has been already successfully applied in many similar applications [8,40,42]. Since our GA actually evolves probabilities of grammar rules, real number coding was chosen for the genotype. The initial population is initialised randomly and then iteratively subjected to evaluation, reproduction, genomic operators and finally succession. These processes are described in more detail at the end of the section. The goodness of grammars induced by the GA is assessed on the basis of its fitting to the training sample of sequences, i.e. the selected grammar is the one which generates maximal log probabilities over the whole set. The algorithm stops when there is no further significant improvement in the score.

The implementation of our grammar induction algorithm is based on Matthew Wall's GAlib library which provides a set of C++ genetic algorithm objects [54].

In order to normalise results regarding sequence length, the objective function is defined as an arithmetic average of logs of probability returned by the parsing algorithm for each positive sample:



where S is the positive sample, W_i _is a sequence from S, G is a given grammar and Pr(W_i_|G) is the probability that W_i _belongs to L(G).

The reproduction step of the GA uses the tournament method which picks randomly a small set of individuals from the original population, and then selects the best one which is added to the set of parents [53]. This is repeated until the appropriate number of parents is chosen. The strength of this method is that the selective pressure is held at the same level during the whole induction process. This strategy with the tournament of 2 competitors was confirmed to be effective in applications similar to ours [8,42]. Once the reproduction step is completed, the population of parents is subjected to standard genomic operations to produce new individuals. We utilised a steady-state scheme with overlapping populations (50%) to assure the stability of the GA algorithm: only the poorer half of the population is substituted by new individuals. Since our GA deals with probabilities of stochastic rules, i.e. real numbers, offspring are produced by crossover where the genetic information of two individuals is averaged. However, some random distortion is added in order to enhance exploratory capabilities of the algorithm (so called blind crossover in Galib's framework). Subsequently, a classical one point mutation operator is used to mutate randomly chosen genes. This allows escaping from evolutionary traps by making possible the exploration of space not covered by parents. The probabilities of crossover and mutation were 0.9 and 0.001 respectively. Finally, the termination condition of the GA is applied: it is based on monitoring the improvement of the score given by the objective function.

Combined with the tournament selection, we apply a scaling scheme exercising diversity pressure on the population, called a sharing function [34], where the fitness score for a given individual is decreased if it is similar to other ones. The application of such a scheme during evolution improves exploratory abilities of the GA by keeping it from settling in local extremes of the fitness landscape. While the Euclidean style distance is an intuitive measure for comparing chromosome probabilities, it suffers from a significant drawback in the case of stochastic grammars where probabilities are normalized. This measure does not take into account the fact that the importance of a given rule depends on other rules with the same LHS non-terminal. To overcome this problem another score, called here Weighted Hamming distance (DistWH), was introduced where comparisons between two chromosomes are also normalized by dividing distances between single genes by the total weight of the chromosomes:



where r_i _and s_i _are values of rule probabilities. Such distance takes into account properties of the normalized grammar without confusing genotype (raw chromosomes) and phenotype stages (after normalisation). As expected, experiments showed Weighted Hamming distance metric is superior both in performance and convergence speed over the Euclidean style measure [55].

### D. Strategies to handle the computational complexity

Although we introduced a strategy to deal with the size of the protein alphabet by dealing with each physiochemical property separately and introducing 3 non-terminal rules which express their quantitative values, the computational complexity of parsing a Context-Free Grammar remains an issue: it is cubic in time and square in space regarding the length of the input sentence. While standard memory resources are adequate for protein sequence scanning (the average length of a sequence is around 350 amino acids), the time complexity is a critical issue in particular during the process of grammar induction. Typically a population made of hundreds of grammars needs to be parsed at each step of the iterative process. Moreover, since the structure of the final grammar is initially unknown, the initial population of grammars should have a set of rules large enough so that it has the potential to express the properties of the pattern of interest.

Most applications reported in the literature use population sizes between 50 and 1000 individuals. Since a compromise had to be found between exploratory capabilities and manageability, we chose to induce grammars using a constant population of 200 individuals. We conducted experiments which revealed that for such a population size, the total number of rules of each individual should not exceed 200 to be able to generate a grammar within a reasonable amount of time (several hours). This constraint led us to design two different strategies regarding the selection of initial rules. The first approach supplies each individual with a complete set of rules associated with random probabilities in order not to introduce any bias in the evolution of grammars. Since we use Chomsky Normal Form for rules, the number of possible rules is bounded by the cube of number of non-terminals. Therefore, in this scheme no more than 7 non-terminals can be introduced to keep the number of rules below 200. The second approach introduces some additional assumptions regarding the structure of the grammar, as described in next section. This allows emphasing the context freeness of the expected solution and increasing the number of non-terminals to 9 without increasing the volume of the initial rule set.

Although genetic algorithms converge whatever their initial population [53], they may not find the global optimal solution. Therefore, for each grammar generation, we actually produced several grammars (usually 3) whose scanning results were combined using arithmetical averaging of scores. Using a cluster of powerful machines - 16 bi-processor 64 bit machines with AMD Opteron™ 244 (1.8 GHz, 1 MB cache) processors - time needed for producing all grammars associated to a given pattern was typically of a few hours.

### E. Detailed description of datasets

The base of the negative samples in all tests consisted of 829 single chain sequences of 300-500 residues returned by PDB30% (accessed on 12th December 2006). Then for each experiment, sequences matching the studied pattern, if any, were removed from the negative set: one sequence was excluded for PS00063 pattern and two for Zinc finger pattern.

• PS00219 is one of two motifs for the anion exchanger family (PDOC00192). The training set consisted of 8 different instances of the 12 residue long PROSITE pattern:

F-G-G-[LIVM](2)-[KR]-D-[LIVM]-[RK]-R-R-Y

and the 11-residue binding site sequence of a protein missed by the pattern:

F-G-G-L-I-L-D-I-K-R-K

We added 76 sequences from the UniProt (9th December 2006) matching PS00219 to the positive part of the testing set.

• PS00063 pattern is one of three PROSITE patterns designed for the aldo-keto reductase family (PDOC00061) binding NAP ligand (Nicotinamide-Adenine-Dinucleotide Phosphate). The pattern is located in the C-terminal of an about 300 residue long protein and centred on a lysine residue which is likely to be the active site residue. The 16 residues long PROSITE consensus pattern is:

[LIVM]-[PAIV]-[KR]-[ST]-{EPQG}-{RFI}-x(2)-R-{SVAF}-x-[GSTAEQK]-[NSL]-x-{LVRI}-[LIVMFA]

The training set consisted of 13 representative instances (chosen on the basis of low sequence similarity) of this pattern and sequences found in the PROSITE false negative set. 86 complete sequences matching the pattern formed the positive testing set. A sequence which contained PS00063 motif was excluded from the negative test set.

• PS00307 is a legume lectin beta-chain signature that binds calcium and manganese located in the C-terminal section of the beta-chain (PDOC00278):

[LIV]-[STAG]-V-[DEQV]-[FLI]-D-[ST]

**Figure 15:**
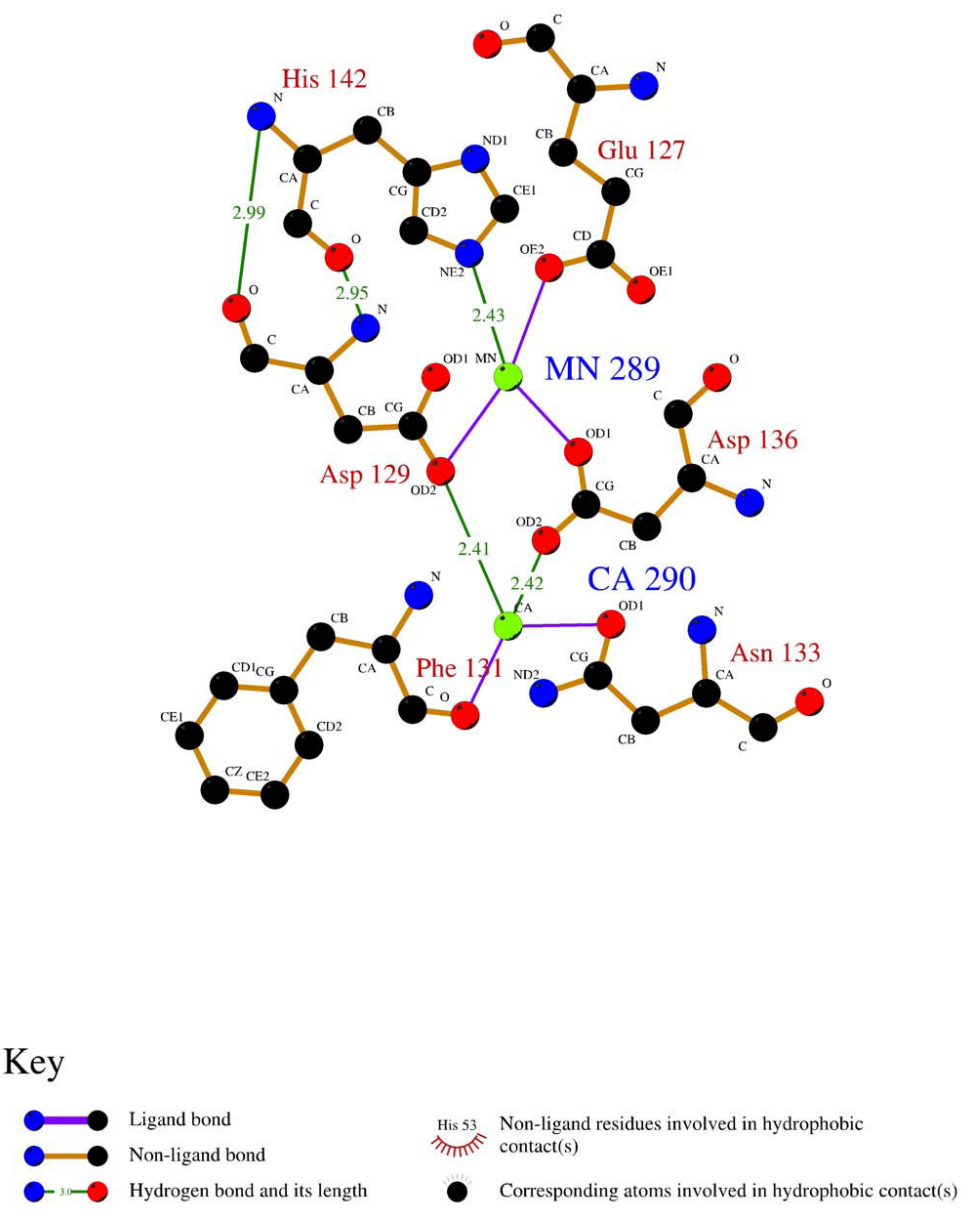
**Ligplot scheme of residues involved in Calcium and Manganese binding in P16404**. P16404: (LEC_ERYCO) Lectin precursor (ECorL) [Erythrina corallodendron (Coral tree)]; PDB: 1AXO[62]}.

The pattern has 64 true positive hits, while 4 sequences are missed and there are 116 false positives. According to the associated Ligplot diagram [59] (Figure 15), many residues taking part in the binding site are not covered by the pattern. Hence, we extended the length of the training sequences up to 25 amino acids, according to multiple sequences alignment (see below). Eventually, the training set consisted of 22 representative sequences of the pattern instance and its neighbourhood (50 residues altogether). Four instances (bold) were much less conserved than the others:



The positive test set consisted of 67 true positive and false negative PROSITE pattern PS00307 matches from UniProt database (accessed on 23rd April 2007).

• The M-phase inducer (MPI) phosphatase family consists of proteins which bind SO4 molecules. The family cannot be covered by a single PROSITE pattern. However, it forms a subset of Rhodanese-like proteins described by Rhodanese domain profile (PS50206). The training set consisted of 16 representative sequences of the binding site and its neighbourhood (total length 25). These short sequences were extracted from the Swiss-Prot database:



The positive test set included 130 non-redundant sequences which belongs to the family according the UniProt (accessed on 22nd February 2008).

**Figure 16:**
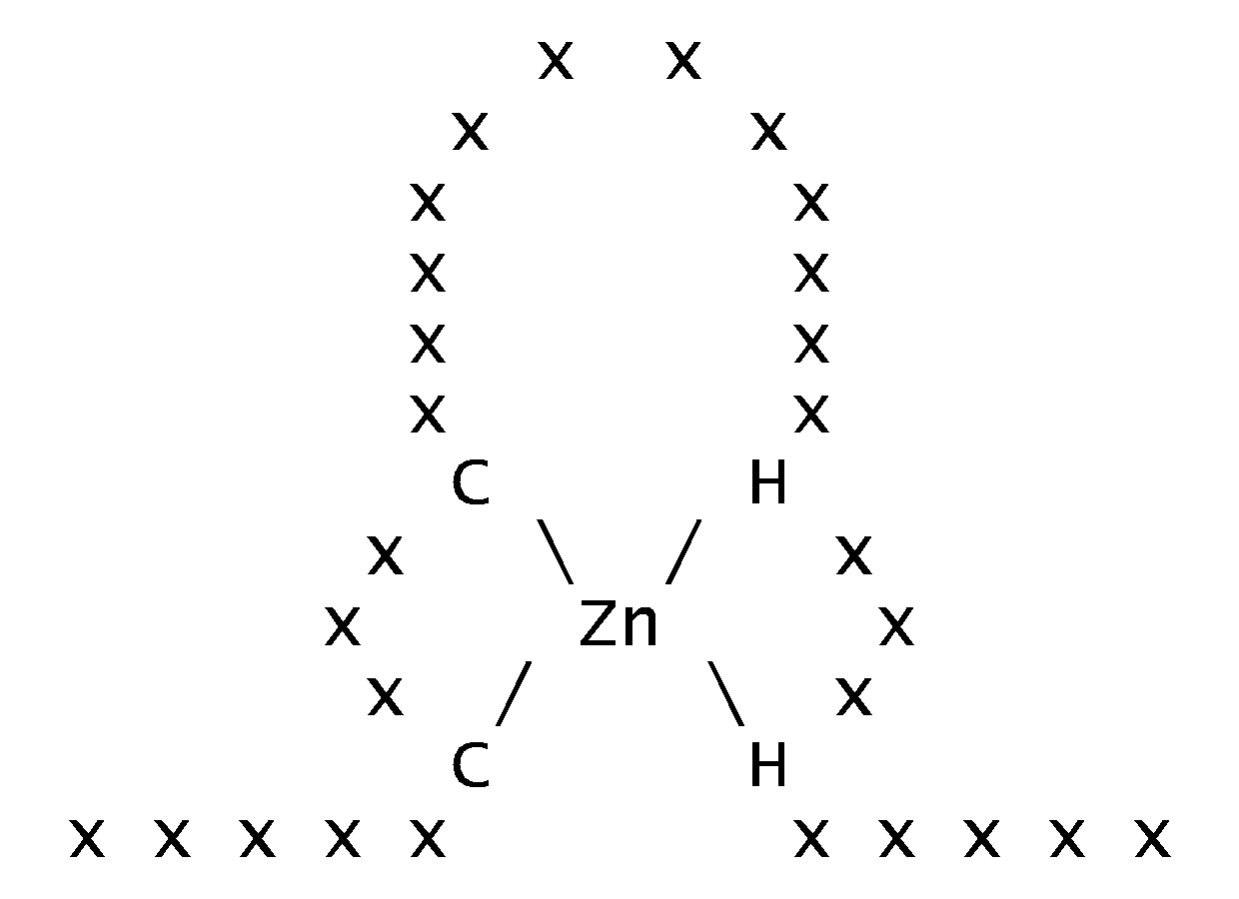
**Typical structure of a zinc finger binding site**.

• Zinc finger is a large superfamily of binding DNA proteins which relies on a zinc atom to support the structure of the binding site (see Figure 16). 11 different PROSITE patterns define their binding sites. We created a zinc finger meta-pattern by selecting all patterns which involved 4 zinc binding residues and did not exceed 30 residues of maximum length.

The 7 patterns that our meta-pattern is based on are the following:

(PS00028)

(PS00518)

(PS00752)

(PS01030)

(PS01102)

(PS13000)

(PS01358)

Lengths of those patterns vary from 10 to 27 amino acids. Also numbers of true positive hits in different sequences in the UniProtKB/Swiss-Prot database differ dramatically between patterns: from 7 (PS00752) to 1598 (PS00028). In order to produce a meta-pattern, a representative set of 20 instances of those patterns was chosen as a positive training set on the basis of 30% or lower similarity of the pattern. The positive test set of 99 sequences was picked randomly from the set of over 500 sequences. Table 8 shows the number of positive training and test samples in both sets according to the pattern type (Notice that some sequences contain instances of more than one pattern from the set). Although none of the PROSITE patterns involved in the Zinc finger meta-pattern was found in the negative dataset, two sequences described in the PDB as containing the Zinc finger motif were excluded from the set.

**Table 8:** Number of positive training and test samples for PROSITE patterns involved in Zinc finger meta-pattern

	**Number of known instances of the pattern**		**Number of known sequences containing the pattern**	
	***Total***	***Training set***	***Total***	***Positive test set***
**PS00028**	10129 (88%)	9 (45%)	1598 (54%)	14 (14%)

**PS00518**	1092 (9%)	1 (5%)	1089 (37%)	32 (31%)

**PS00752**	7 (<<1%)	1 (5%)	7 (<<1%)	0 (0%)

**PS01030**	28 (<<1%)	4 (20%)	28 (1%)	3 (3%)

**PS01102**	20 (<<1%)	1 (5%)	20 (1%)	3 (3%)

**PS01300**	169 (1%)	3 (15%)	169 (6%)	22 (22%)

**PS01358**	117 (1%)	1 (5%)	73 (2%)	28 (27%)

**Total**	11562 (100%)	20 (100%)	2984* (100%)	102* (100%)

*Some sequences contain instances of more than one pattern from the set.
